# Cisgender Women Navigating Genital Insecurities: Coping, Genital Cosmetic Surgery, and the Role of Medical Encounters

**DOI:** 10.1007/s10508-025-03322-z

**Published:** 2025-12-09

**Authors:** Hannelore Van Bavel

**Affiliations:** Rhea Research Centre for Gender, Diversity and Intersectionality, Vrije Universiteit Brussel, Pleinlaan 2, 1050 Brussels, Belgium

**Keywords:** Genital self-image, Body image, Female genital cosmetic surgery, Labiaplasty, Medicalization, Genital insecurity

## Abstract

This study explored how cisgender women in Belgium experience and navigate genital self-image. Drawing on seven focus group discussions with cisgender women from the general population (n = 34) and six in-depth interviews with cisgender women who had undergone labiaplasty, the research situates surgical decisions within a broader continuum of responses to genital insecurity. Across both groups, participants described such insecurity as widespread. While many found reassurance over time—through sexual experience, peer conversations, or exposure to representations of vulval diversity—others sought to reshape their relationship with their bodies through body-positive art projects or surgery. Most interviewees described medical encounters in which clinicians validated their concerns and presented surgery as a means of correction, though one woman recounted a contrasting experience involving reassurance, information, and a mandatory reflection period. Although all interviewees expressed satisfaction with the outcome, many also reflected critically on the cultural and emotional pressures that informed their decisions. The findings underscore the need for comprehensive sex education, cultural interventions that affirm genital diversity, and improved clinical training to support informed, confident, and autonomous decision-making.

## Introduction

Genital self-image encompasses an individual’s attitudes, feelings, and behaviors surrounding their own genitals (Sharp & McGrath, 2025). Concerns about genital appearance have been documented across cisgender, transgender, and intersex populations and may relate to features such as size, symmetry, shape, color, hair, odor, or perceived cleanliness (ibid). Poor genital self-image often arises when people perceive their own genitalia as deviating from a dominant sociocultural ideal. For cisgender women in contemporary Western contexts, this ideal emphasizes a smooth, hairless vulva with the inner labia concealed by the outer (Sharp et al., 2016). Such ideals are not universal; in parts of Uganda and Mozambique, for instance, elongated labia are culturally valued (Martínez Pérez et al., 2016). The current Western vulval beauty ideal has been shaped by a long history of racist and sexist medical discourse. From the nineteenth century onward, longer labia were increasingly associated with sexual activity: They were considered normal, though unattractive, in older married women, but suspicious in young, unmarried women. They were also constructed as a marker of Black women’s supposed hypersexuality; when observed in white women, elongated labia were pathologized as signs of promiscuity or even racial degeneration (Nurka, 2018). More broadly, this vulva ideal reflects a cultural logic in which women’s bodies have long been framed as leaky, excessive, and polluting (Douglas, 2002). Within this frame, the “neat,” hairless vulva embodies control and hygiene, disciplining what is otherwise imagined as unruly or excessive flesh.

This historically constructed ideal continues to shape perceptions today. Pornography, social media, and even medical textbooks typically present only a single normative vulval appearance, thereby erasing diversity and reinforcing a narrow aesthetic (e.g., Bordo, 2009; Holliday, 2020; Schick et al., 2011; Sharp et al., 2016). Repeated exposure to such representations has been shown to negatively affect cisgender women’s genital self-image and to narrow their perceptions of what constitutes a “normal” vulva (e.g., Howarth et al., 2016; Moran & Lee, 2014; Schick et al., 2011; Sharp et al., 2016). A poor genital self-image has been linked to a range of negative outcomes, including reduced sexual confidence, diminished pleasure, lower self-esteem, and even avoidance of medical care such as gynecological check-ups (e.g., Dias Leite et al., 2025; Vigil et al., 2021).

Feminist scholars of beauty have argued that the increasing scrutiny of the vulva exemplifies both the intensification and extensification of the beauty regime, whereby ever more areas of the body are drawn into practices of self-surveillance and modification (Gill, 2021). What was once considered a private or invisible part of the body is now evaluated against narrow aesthetic norms, subjected to grooming, and even surgically altered to achieve a “neater” look. Within the current neoliberal ideal of self-management and responsibilization, women are positioned as responsible for continually optimizing their bodies (Widdows, 2018). In this framing, cosmetic genital procedures are marketed as responsible self-care and bodily maintenance.

At the same time, Braun (2019) argued that the dominant vulval ideal has become increasingly medicalized. A specific aesthetic is now presented as “normal” and “healthy,” while natural variations are pathologized as “abnormal.” For example, longer inner labia are labeled as “labial hypertrophy,” a term that suggests medical abnormality. This medicalization enables surgeons to reframe aesthetic procedures as corrective interventions. These neoliberal and medicalization framings reinforce each other, positioning genital cosmetic surgery not merely as a choice, but as a responsible and even necessary response to bodily deviation.

This process of medicalization is facilitated by a broader cultural silence around genital diversity. Because vulvas are rarely discussed, depicted, or compared in everyday life or sex education, many people grow up without a clear sense of what variation looks like. In the absence of exposure to diverse vulvas, the narrow aesthetic ideal is mistaken for biological normativity. Medicine then steps in as an authority that appears to confirm these assumptions, lending clinical legitimacy to what is essentially a socially produced standard. In this way, silence and limited knowledge do not simply leave people uninformed; they create the conditions in which aesthetic preferences can be recast as medical concerns.

This ideal also impacts the attitudes and advice of medical professionals. Trained with the same narrow anatomical images and diagnostic terms, they too may interpret diversity as pathology. Studies furthermore show these judgments are shaped by gender and specialty: Reitsma et al. (2011) found that male plastic surgeons were especially likely to recommend labiaplasty and rated smaller labia as more “natural” and “attractive.” Across specialties, male physicians were more inclined to suggest surgery than female colleagues, suggesting that personal aesthetic preferences and gendered ideals of normality can shape medical decisions.

When it comes to how women deal with genital insecurities, the literature mainly focuses on two main coping mechanisms. On one end of the spectrum are forms of avoidance, such as diminished sexual confidence, reduced pleasure, or withdrawal from gynecological care (Dias Leite et al., 2025; Vigil et al., 2021). On the other end is active modification of the body through female genital cosmetic procedures (FGCP). These encompass both surgical and non-surgical interventions that alter the appearance or function of the vulva or vagina of cisgender women in the absence of medical necessity, including labiaplasty, clitoral hood reduction, and vaginal tightening, as well as non-surgical treatments such as intimate bleaching, laser vaginal tightening, and the so-called “G-spot shot” (filler injections) (Kavouni & Creighton, 2019).^1^

But between avoidance and surgery lies a broader spectrum of coping that remains underexplored. Little is known about how women who do not pursue surgery nonetheless navigate genital insecurities. FGCP patients are often studied in isolation, leaving the wider social and emotional spectrum—and its connections to gender, beauty, and medicine—poorly understood. As a result, those who seek surgery may be treated as exceptional, rather than as situated within a broader field of normative cultural pressures and bodily self-evaluation. To address this gap, this study asks: How do cisgender women navigate genital insecurities across the life course, and what coping strategies do they employ? What, if any, role do medical professionals play in this process?

## Method

### Participants

This study used qualitative methods, including seven focus group discussions (FGD), six in-depth interviews, and participant observation. It was conducted in Belgium, with participants recruited from Brussels and Flanders. Belgium has relatively liberal sexual attitudes that are comparable to those found in other Western European contexts.

The FGDs involved 34 women aged 18 to 72 from the general population, with no selection based on genital self-image, as the aim was to explore a wide spectrum of experiences. To capture potential differences across the life course, I organized five groups by age bracket (18–20, 21–30, 30–50, and two groups of 50 +). In addition, I organized one group for recent mothers, recognizing that pregnancy and childbirth can directly shape women’s experiences of their genitalia. Finally, one group was held in English to accommodate non-Dutch speakers living in Belgium (Table 1). Group sizes were kept small (4–7 participants) to foster open discussion on a sensitive topic.

**Table 1:** Overview of focus groups

Focus group	Target group	Age range	Language
FGD1	Young adults	18–20 years old	Dutch
FGD2	Adults in their 20s	21–30 years old	Dutch
FGD3	Gave birth in the last 12 months	26–39 years old	Dutch
FGD4	Adults in their 30s and 40s	30–50 years old	Dutch
FGD5	Non-Dutch-speaking women living in Belgium	26–37 years old	English
FGD6	Women over 50	51–53 years old	Dutch
FGD7	Additional 50 + group (due to small size of FGD6)	53–72 years old	Dutch

### Measures and Procedure

The six interviews were conducted with women aged 19 to 36 who had undergone labiaplasty (Table 2). This separate interview format was chosen because FGCP patients generally felt uncomfortable disclosing their FGCP status in a group setting.

**Table 2:** Overview of participants who have undergone labiaplasty

Participant	Current age	Age at labiaplasty
P1	36	25
P2	19	18
P3	32	32
P4	22	15
P5	36	36
P6	25	16

I also participated in two art projects engaging with genital self-image: the Vulvarium project (plaster vulva casts by Austrian artist Viktoria Krug) and Vulva Obscura (photographic portraits by Belgian photographer Hanne Lamon). I observed women as they took part and listened to their conversations with the artist about their vulvas and their reasons for participating.

Recruitment for focus groups was done via flyers describing the study as an exploration of women’s perceptions and experiences of their genitalia. These were circulated on social media (including the project website, www.[name].com) and in public spaces such as universities and community centers. A separate flyer specifically invited women who had undergone FGCP. This was also shared on social media and through plastic surgeons. Four FGCP participants responded via [name] (www.[name].com; [name]_ on Instagram), one was referred by a plastic surgeon, and one via photographer Hanne Lamon. The overall sample aimed to balance diversity and depth, with data collection ending once no substantially new themes emerged.

The focus group format was inspired by 1970s feminist women’s health groups, which aimed to help women reclaim knowledge and bodily autonomy from the authority of male medical professionals. The focus groups included a participatory element: participants were invited to paint a vulva, inspired by GeetLush’s feminist paint-alongs (Geetlush, 2023; Nichols, 2000). The aim was to facilitate natural conversation around a sensitive topic by offering a visual and tactile activity (Hartley et al., 2023). Painting allowed for moments of silence or disengagement, as participants could simply concentrate on the task at hand. Two groups opted out: recent mothers (for practical reasons, as they brought babies along) and older women (who preferred informal conversation over drinks). Focus group discussions lasted on average two hours; interviews about one hour.

### Data Analysis

Written informed consent was obtained from all participants prior to their involvement in focus groups, interviews, or participant observation. All sessions were audio-recorded with participants’ explicit written consent, and participant observation was documented in fieldnotes. Recordings were transcribed verbatim and pseudonymized, and both the transcripts and fieldnotes were analyzed using reflexive thematic analysis, a method for interpreting patterns of meaning in qualitative data through a process of iterative coding and reflexive engagement by the researcher (Braun & Clarke, 2021). The coded data were audited by a colleague from the research center, who offered an external perspective and posed questions that helped further refine and deepen my interpretations.

All interviews and focus groups were in Dutch, except for one focus group held in English. All quotes presented in this paper were translated by the author. Focus group participants are referred to by pseudonym, age, and focus group number–for example, (Jolien, 39, FGD3). Interviewees who had undergone labiaplasty are referred to by a participant number and age–for example, (P1, 36).

## Results

This section explores how participants experienced and made sense of genital insecurities, the strategies they used to navigate these feelings, and the role of medical encounters in shaping their responses. I begin by outlining the nature and perceived sources of insecurity. I then trace a continuum of coping strategies, from silence and avoidance to confidence-building, peer conversations, and participation in art projects. Surgery appears within this spectrum as one—but not the only—response. The next section considers medical encounters, which could reinforce or even generate insecurity, though in rare cases offered meaningful reassurance. Finally, I examine how women who underwent labiaplasty reflected on the outcomes, revealing both satisfaction and ambivalence, as well as lingering doubts and shame despite surgical change.

### Genital Insecurities

#### Forms of Insecurity

Across focus groups and interviews, most participants shared that they–at least at some point in their lives, if not now–had felt insecure about their own vulvas, and believed that such feelings were widespread. When asked how women generally feel about their genitalia, a participant reflected, “Strangely, when I saw the topic, I automatically thought of insecurities, of people feeling bad about their vulva” (Elisabeth, 31, FGD5).

Participants in the oldest focus groups (50 +) described their generation as largely disconnected from their genitalia, marked less by insecurity than by a lack of awareness. Vulvas, they said, were rarely seen or discussed. In contrast, they felt younger generations were far more conscious of, and concerned about, genital appearance, a shift they attributed to the increased visibility of idealized vulvas in pornography and online media. A 57-year-old participant (Christiane) said she felt “from another planet” when she first heard women discussing labiaplasty at the hairdresser’s. This suggests that it is not limited knowledge alone that drives insecurity, but the combination of insufficient knowledge with intensified genital ideals, a dynamic particularly affecting younger generations.

Across the focus groups and interviews, women raised a variety of concerns related to their genitalia, which can broadly be grouped into appearance-based and function or hygiene-related anxieties. Appearance-related concerns centered on the size, symmetry, and visibility of the (inner) labia, the shape of the mons pubis (“venus hill”), pigmentation, and vaginal tightness. Pubic hair was discussed not only as an aesthetic concern but also as a source of deeper gendered anxiety. While many participants mentioned feeling pressure to groom their pubic area to meet contemporary beauty standards, several also expressed shame about hair in areas like the anus—regions where hair was not just considered unattractive, but fundamentally unfeminine. Functional and hygiene-related concerns included odor, discharge, and vaginal infections.

Many said genital insecurities began during adolescence—a time of rapid physical change and heightened self-awareness—recalling confusion or discomfort about the growth of pubic hair or seeing their vulva for the first time. These feelings often intensified in anticipation of sexual encounters, whether with a first partner or a new one. For some, childbirth brought fresh concerns about tearing, loss of vaginal tightness, and lasting changes. Menopause was also said to bring renewed concerns related to aging, such as dryness or changes in appearance.

#### Sources of Insecurities

Participants often attributed their genital insecurities to a narrow, idealized beauty standard, which some compared to a “childlike vulva, where the outer labia always neatly enclose everything” (Tine, 34, FGD3). This ideal was said to be learned from various channels, particularly pornography and social media, where edited and curated bodies made participants question their own appearance: “On Instagram, everything looks smooth and flat, and then you look at yourself and think: is mine weird?” (Fien, 19, FGD1).

Advertisements for cosmetic procedures were said to introduce new insecurities: “You see labiaplasty everywhere now, and it makes you think: is mine okay?” (Sofie, 46, FGD4).

“[I heard about] anal bleaching, and I was like, ‘Oh my God, should I be concerned about that?” (Giulia, 36, FGD4).

Beauty norms were also reinforced through small, everyday interactions. Lien (33, FGD4) noted that communal showers at the gym made her aware that “everyone else is shaving,” which led her to wonder: “Oh, maybe I should do that too?” Robin similarly described how a passing comment from her sister made her start shaving: “[My older sister] would say: ‘When I shave, I get these bumps.’ And then you start thinking: ‘I should start shaving too!’ So it’s not like anyone tells you outright that you have to shave, but you pick it up subtly, from little comments here and there. And I think you really internalize that, especially because it’s linked to shame.” (Robin, 26, FGD2).

Beyond beauty ideals, participants described how female genitalia are often framed as dirty, smelly, or in need of control. Claire (37, FGD5) recalled how “she smells of fish” was a common insult, reflecting a broader cultural association between vaginas and unpleasant odor. These messages were reinforced by marketing for feminine hygiene products—douches, wipes, and perfumed pads—that implied vulvas require constant “maintenance” to be acceptable: “On TikTok, you see all these vaginal washes and products that make you think you need them” (Lien, 33, FGD4).

In addition to beauty standards and negative cultural messages, participants described how limited sex education, minimal exposure to diverse bodies, and a broader culture of silence contributed to genital insecurity. Across all age groups, they noted that a lack of open conversation and clear information left them uncertain about what was normal, fostering confusion, shame, and self-doubt.

Sex education was described as brief, heteronormative, and focused mainly on reproduction, typically excluding topics like pleasure, consent, and genital variation. Several participants recalled that even basic anatomy was skimmed over or shown only in idealized, clinical drawings. The clitoris, in particular, was rarely mentioned: “We only learned about the clitoris from [a women’s] magazine, not school” (Jolien, 39, FGD3).

Without accurate knowledge or language, participants said it was difficult to voice concerns or seek support. Some also noted that Dutch terminology reinforces shame: “Everything starts with ‘schaam’ [shame] in Dutch: schaamlippen [shame lips, i.e. labia], schaamstreek [shame area, i.e. pubic area]. It teaches you from the start to feel embarrassed.”^2^ (Nina, 22, FGD2).

These knowledge gaps were reinforced by silence at home. Parents often avoided discussions about sex, menstruation, or genitalia. Some participants were given books rather than direct conversations; others turned to friends or the internet. Two participants from households with a Moroccan migration background described additional cultural taboos around discussing sexuality, especially with male relatives: “In our culture, you don’t talk to your dad or uncle about these things. It would be rude to even mention your vagina” (Noor, 19, FGD1).

Several women recalled never seeing real naked female bodies growing up. While some who grew up in naturist or open households felt more at ease early on, even they experienced shame by adolescence. Nonetheless, participants stressed that beyond anatomical knowledge, exposure to real, diverse adult bodies—not just airbrushed or clinical images—was essential for developing a healthy self-image. Without visual references, coupled with silence and shame, many were left unsure of what was normal.

While beauty ideals are absorbed through general socialization – via family, peers, and media – participants described a distinct and especially potent layer of insecurity tied to sexual desirability. Comments from men, whether about their own genitalia or women’s genitalia in general, were experienced not simply as casual remarks but as signals of sexual worth, positioning women within a perceived hierarchy of desirability. Many participants recalled hearing mocking or critical remarks throughout their lives, often framed as jokes but conveying a clear message: certain vulvas are undesirable.“You had the boys who would say things like, ‘She’s got a loose pussy,’ or, ‘That one’s loose down there’” (Noor, 19, FGD1).

Such comments appeared not only in adolescence but continued into adulthood, surfacing in casual conversations among male peers and even in professional settings: “[At university, boys] would say things like, ‘She had so much pubic hair,’ or, ‘She had really big labia.’ That’s when I started wondering: ‘Wait, I’ve never thought about this before, but am I perhaps abnormal too? Are they going to talk about me like that after [having sex with me]?’” (Nina, 22, FGD2).

“A male colleague once said about an article in [a women’s magazine] on vulvas: ‘Well, if you had such long labia, of course you would just get surgery, right?’ I felt so exposed. I thought: are mine not okay then?” (Jolien, 39, FGD3).

Beyond general remarks about female genitalia, many participants also recalled receiving direct comments about their own bodies. Some of these were openly hurtful—for example, one participant’s first boyfriend bluntly told her, after giving oral sex: “You stink.” The comment left her deeply anxious about receiving oral sex in the future. While such overt rudeness may reflect the speaker’s immaturity or lack of empathy, participants also described how less obviously cruel—or even well-intentioned—remarks could leave lasting effects. One woman in her late 30 s (Sofie) recalled a casual bedpartner reassuring her that she was “not yet loose down there,” implying that women’s vaginas might become “loose” with age. She had never been concerned about vaginal tightness before, but this comment made her wonder whether men were constantly evaluating her in that way. Even casual reassurances, participants noted, can reveal and reinforce unspoken standards.

This imagined male gaze influenced grooming, self-perception, and feelings of adequacy. Some participants were surprised, however, to find that their assumptions didn’t always match reality: “Actually, I don’t think I ever had a partner who asked me to shave. I just assumed they preferred it that way” (Monica, 31, FGD5).

#### Consequences of Insecurities

Participants described how failing to meet certain expectations, especially around grooming or appearance, left them feeling unattractive, abnormal, or even “dirty.” Some participants spoke of anxiety, self-criticism, and a self-image shaped by how closely their bodies matched idealized standards. “It’s not realistic, but you see these things at such a young age, especially on social media. It really damages your self-image” (Fien, 19, FGD1).

Beyond low self-esteem, many participants described a deeper sense of disconnection from their bodies, particularly their genitalia. They spoke of growing up with the idea that their bodies existed not for themselves, but to be looked at, evaluated, and desired: “It’s like this [gestures to vulva] was made for a man. We never think of it as ours.” (Lina, 19, FGD1).

Some described how their sense of self-worth became tightly bound to desirability: “It showed us exactly how we should be for them to like us. That really shaped how I saw myself.” (Monica, 31, FGD5).

This feeling of being on display often led to a kind of estrangement, a sense of unfamiliarity or discomfort with one’s own body. Rita said that she only looked at her vulva when worried something was wrong; even then, the experience could feel alienating: “It was like looking at something that didn’t belong to me.” (Rita, 51, FGD6).

Rather than something to inhabit or enjoy, the vulva was often experienced as something to manage, conceal, or worry about.

Genital insecurities often shaped how participants moved through the world, especially in situations involving nudity. Several women described feeling held back in daily life, less free to act on impulse or enjoy certain experiences: “It creates a kind of hesitation in how you act… You trust yourself less. You don’t follow your intuition” (Liliane, 51, FGD6)

Many avoided swimming, saunas, or communal changing rooms out of discomfort about their vulva or visible body hair: “If I haven’t waxed, I’ll ask my partner to take the kids swimming. Otherwise, I just don’t go.” (Claire, 37, FGD5).

“People would talk about skinny dipping on holiday, and then I would think: ‘Shit, I want to do that too, but I don’t dare to because [I am uncomfortable] showing my vulva.” (Charlotte, 30, FGD3).

“I wore sanitary pads just so my labia wouldn’t show through my gym shorts.” (Robin, 26, FGD2).

This discomfort extended to medical settings as well. Many participants described gynecological exams as invasive and embarrassing, with some avoiding them altogether or requesting female practitioners. For most, the discomfort was linked to cultural norms about modesty and privacy, rather than concerns about appearance. However, for some, insecurities about looking “normal” added a further layer of anxiety.

Many participants said genital self-consciousness made it difficult to fully enjoy sex. Anxiety about body hair, smell, and appearance often led to stressful preparation and constant preoccupation. Rather than focusing on pleasure, they worried about looking clean and desirable.

“The stress when you’re dating someone new: ‘I still have to shave, I definitely have to wash, because if it smells, that’s the worst thing ever!’ On some level, it’s just normal that you have to be hygienic for your partner, but on another level, it’s also just hair – is that really unhygienic? Yet, I often feel unhygienic if I haven’t shaved” (Robin, 26, FGD2).

This constant self-monitoring made it hard to relax or be present in the moment. Oral sex felt especially exposing, as it placed focus on a part of the body many already felt insecure about.

“With oral sex […], suddenly all the attention is on your genitals. I’ve always really struggled with that – letting go of the shame and actually enjoying it, because I am constantly preoccupied with, I don’t know, judging myself” (Yente, 23, FGD2).

Instead of tuning into their own pleasure, participants often felt they were performing what sex should look like—adjusting their bodies, facial expressions, and sounds to meet imagined expectations. One woman described her thoughts during sex:

“Oops wait, I need to arch my back a bit more, I need to pull in my stomach, because oh no, he’s looking… but oh, I also shouldn’t make weird faces, so I have to try to do it like this, and like that…” (Noor, 19, FGD1).

While this quote focuses on sexual performance more broadly, participants’ accounts suggest that genital insecurity was woven into these dynamics of desirability. Even when not explicitly about the vulva, preoccupations with looking “right” and meeting imagined standards were deeply tied to the broader experience of feeling their bodies—including their genitals—were under scrutiny. This shaped sexual dynamics overall: feeling unattractive or ashamed often led women to focus on being accepted and desired, making it feel safer to perform desirability or comply with partners’ preferences rather than express their own. Echoing earlier themes of alienation, when women internalized the belief that their bodies existed to be groomed and evaluated by others, it became harder to set boundaries or prioritize their own desires. Several said they were so focused on being liked that they lost touch with their own preferences. In this context, they questioned whether they could say no without jeopardizing their partner’s approval:

“If I say yes to sex, does that mean I have to be okay with everything? What if I say no to something, and he thinks I’m weird or doesn’t want me anymore?” (Noor, 19, FGD1).

### Coping with Genital Insecurities

#### Growing Confidence Over Time

Most participants said they did not actively address their genital insecurities. Instead, they kept their feelings to themselves:

“I really just kept it to myself for a long time. I think this is something that’s often just swept under the rug, like most body insecurities. I think it’s often internalized quietly” (Yente, 23, FGD2).

However, many women said their insecurities faded naturally with age, life experience, and the realization that others shared similar concerns.

“The older you get, the better you understand the norms that exist in society […]. You also talk more with friends, your mum, your sister–and realize they’ve been through similar things” (Robin, 26, FGD2).

Sexual experience played a significant role in reshaping their perceptions. For some, it was the realization that men cared less about genital appearance and pubic hair than they had expected. Others found that affirming comments, intimacy on their own terms, or simply engaging in sex more frequently helped build a stronger sense of bodily confidence.

“I had a long-term partner who would sometimes look at me in the mirror and say, ‘Look, this looks amazing.’ And I would see myself, too. That really helped… I had never really looked at my genitals before” (Elisabeth, 31, FGD5).

Queer participants, all of whom had previously had sex with men, described feeling more at ease in sexual relationships with women. They spoke of experiencing less pressure, more acceptance, and greater appreciation for bodily diversity. Some linked this to the shared experience of having a vulva, while others noted that queer spaces more broadly fostered positive views of vulvas:

“Among queer women, vulvas are talked about more, and seen as beautiful. Diversity is celebrated. I know people who make vulva cupcakes as an activity…” (Robin, 25, FGD2).

For others, gaining knowledge also played a key role. Reading about anatomy, seeing medical illustrations, or exploring body-positive resources helped reduce the sense that something was wrong with them. Several participants mentioned the impact of encountering media and art projects that celebrated vulval diversity. Initiatives like The Vulva Gallery—watercolor portraits of diverse vulvas by artist Sam Hil Atalanta—offered representations they had never seen before, and helped them place their own appearance within a wider, more accepting frame.

“I don’t even remember where I came across it–maybe the Vulva Gallery on Instagram or some other website. It just had drawings of vulvas. And I thought, oh wow, okay. I mean, I’ve never even seen my sister’s vulva, even though we’re close. But just finding those references online helped” (Tine, 34, FGD3).

#### Reclaiming Through Art

Some women made intentional efforts to reclaim a positive relationship with their vulvas through artistic projects that encouraged curiosity, acceptance, or even celebration. I attended three sessions where women had their vulvas cast in plaster by Austrian artist Viktoria Krug as part of the Vulvarium project. Each had previously struggled with insecurity, but they saw the casting process as a way to honor or even reclaim their genitalia, transforming something once uncomfortable into art to be proud of.

"As a child, I just saw it as one thing; it was my vulva. I never labelled or judged it; it was just mine. It’s only because of other people’s comments–like ‘oh, she’s really present there’ or ‘what a bush you have’–that I started to feel insecure. Seeing my own vulva as a statue really changed something. You never normally see it from that perspective. It almost felt like it belonged to a sister, not me, and that made it easier to be kind to it, to see its beauty. That has definitely changed how I feel about it" (Jana, 37).

Similarly, participants in Hanne Lamon’s Vulva Obscura photography project, which captured vulvas through a camera obscura and Polaroids, described the experience as transformative. By seeing their vulvas represented in artistic photography, they were able to reframe their bodies outside of traditional beauty standards or pornographic ideals, recognizing them as just one of many unique, normal variations.

#### Seeking Change Through Surgery

Some women sought to improve their relationship with their genitalia—and their self-esteem—through surgery. Notably, their accounts echoed many of the same themes raised by participants who did not undergo labiaplasty: comments, comparisons with others, and the fear of judgment by sexual partners. Like many focus group participants, P4, who underwent labiaplasty at age 15, described early comparisons with her mother and sister and fears about potential future sexual partners. She explained:

“I was fifteen, and I had just started looking at myself. I became very insecure because it really bothered me. I was beginning to explore dating, not sexually yet, but still. I remember thinking: if I ever do have sex, this will be a huge insecurity unless I fix it. […] I was too afraid to start a relationship. Too afraid of any sexual contact. I was young, but still, I really didn’t dare” (P4, 23).

As also described by focus group participants, remarks from others played a powerful role in shaping perceptions. For P3, her decision was triggered by an external comment, although her initial discomfort was not purely aesthetic. She had always felt uneasy about her vulva, but the issue intensified after a male acquaintance casually remarked that his bed partner’s labia looked like “slices of ham”:

“That really stuck with me. I thought: maybe someone would say that about me too. I’d never really considered surgery before, even though I’d sometimes felt discomfort. But after that, I started focusing on it, and maybe even feeling more ‘discomfort’–between inverted commas – than before” (P3, 32).

In the focus groups, some women said that positive sexual experiences had helped improve their genital self-image. However, P2 and P6’s experiences show that even supportive partners did not always alleviate deep-seated insecurities. P2 underwent labiaplasty at 18, and P6 at 16. Unlike P4, both were already sexually active and said their partners had never criticized their vulvas, yet they still felt deeply insecure:

"From the age of 13, I had a negative self-image of my labia, which were one centimeter longer. It bothered me every day. No bed partner ever had a problem with it, and some also didn’t understand why I would undergo the procedure. The mental problem was in me" (P2, 19).

P1’s experience also highlighted how sexual encounters can worsen genital insecurities. She first noticed her asymmetrical labia as a teenager, but it wasn’t until her early twenties, when she became more sexually active, that discomfort began to take hold. What initially struck her as unusual became a source of shame when partners struggled to locate her clitoris, mistaking her labia for it:

“I became more sexually active and noticed that when men tried to finger me, they would often mistake my inner labia for my clitoris. I was too embarrassed to say, ‘You’re not in the right spot, that’s just my labia.’ That’s when I started wondering: is this even normal?” (P1, 36).

Similarly, while P5 had experienced physical discomfort, especially during long bike rides and horseback riding, it wasn’t until a new sexual partner commented that he had never seen labia as long as hers that she began to rethink her body:

"I had a new bed partner who spoke to me about it, and his comment really made me reflect on it. He said, ‘Wow, that’s really long, I’ve never seen that before’" (P5, 36).

While she noted that sex could be uncomfortable and that she sometimes had to spread her labia to “make room” for penetration, she added, “You get so used to your body.” She explained that during sex, her labia would sometimes slip inside, causing significant pain:

“And that, combined with the discomfort when biking or horseback riding… that’s when I started looking it up: ‘Yeah, actually, it’s possible to operate this.’ And then I thought: You know what, I’m going to do this for myself” (P5, 36).

Remarkably, five out of six women mentioned some degree of physical discomfort, but their accounts revealed that surgery decisions were rarely about discomfort alone. P1, for example, noted some pain while cycling but estimated that 80% of her motivation was shame. P6 similarly reflected:

"Physically, I did notice sometimes on the bike that it was a bit uncomfortable. But looking back now, I don’t think that it was to the extent that surgery was really necessary. It was more psychological, like: oh no, I have a strange shape or something, not comparable to others” (P6, 25).

P3 and P4 also mentioned discomfort but pointed to key moments when external comments tipped the balance: P3 recalling a man’s remark about another woman’s labia looking like “slices of ham,” and P5 receiving direct comments about her own vulva. These stories show how physical discomfort was often entangled with deeper feelings of shame and insecurity. While bodily sensations mattered, they were rarely the sole motivator; decisions were shaped by a mix of embodied experiences, internalized beauty ideals, and external validation or judgment.

### Genital Insecurities in Medical Encounters

Some participants who felt insecure about their genitalia nonetheless didn’t dare raise the issue with medical professionals to ask whether their appearance was normal:

“Honestly, I don’t find it difficult to have a gynecological exam. I mean, they’re gynecologists, they see that a hundred times. That’s not weird to me. But… I’ve never dared to ask: ‘Is this actually normal? Do most people look like this?’” (Sofie, 46, FGD4).

Perhaps most concerning were cases where medical professionals themselves introduced insecurities, even when participants hadn’t been worried. Charlotte (30, FGD3) described how, during her first gynecological appointment as a teenager, the doctor pointed to her inner labia and said, “If you want, we can operate on that.” She had never expressed discomfort, nor seen her labia as a problem, until that moment. “I didn’t even know it was something you could be insecure about,” she recalled.

Participants who did bring up concerns often described interactions that reinforced, rather than alleviated, their worries. Lucia (34, FGD5) consulted a male gynecologist about a small mole on her labium. Though she mainly wanted reassurance, he recommended removal – not for medical reasons but because, he said, “a man who sees it might think you’re sick.” Reflecting on the experience, Lucia said: “If I had been younger, I think I would have believed him.”

Similarly, 5 out of 6 women who underwent labiaplasty reported that when they raised their insecurities, clinicians responded with medicalized language that framed their vulva as problematic. Terms like “problem,” “labial hypertrophy,” or “excess skin” positioned natural variation as something needing correction. For example, P2 first voiced her concerns to a female gynecologist, who remarked that her labia were indeed “notably longer,” reinforcing her sense of abnormality. The gynecologist referred her to a plastic surgeon, noting that surgery might also be needed on the clitoral hood, which she couldn’t perform herself. When R2 met the plastic surgeon, he quickly identified a “problem” and moved straight to discussing the surgical details, without offering non-surgical options or asking whether she had any doubts:

“He looked and felt briefly, and immediately pointed out the ‘problem,’ saying, ‘Yes, yes, there is some excess skin.’ He quickly moved on to the practical details of the procedure and never asked if I doubted” (P2, 19).

P4 recounted a similar interaction:

“The doctor looked and said, ‘Yes, I can see why this would make you feel insecure. It’s possible to [remove it]’” (P4, 23).

P1 recalled asking her gynecologist if her labia were normal. Her gynecologist responded that the asymmetry was something they “saw often,” but didn’t explicitly say it was normal. When P1 asked whether anything could be done about it, she replied casually, explaining that it could simply be cut off – a quick 20-min procedure that would cost only a few euros. This matter-of-fact tone gave P1 the impression that labiaplasty was not only possible but common and expected:

“And that’s when I thought: wow, if it’s that easy and routine, then other women must be doing this too. I can’t be the only one” (P1, 36).

In half of the cases (P2, P3, P5), surgeons also recommended a clitoral hood reduction—something the patients had not initially asked for. Notably, this was the case even for P3, who said she sought labiaplasty for discomfort and was not concerned about aesthetics. Nonetheless, she was told by her surgeon that if only her labia were reduced, her clitoris might “look like a little penis” This remark, she said, planted aesthetic concerns she hadn’t had before.

In the case of P5, during the physical examination, her plastic surgeon suddenly asked if she had difficulty climaxing. She replied that she sometimes struggled to climax with partners but never on her own. The surgeon suggested that the difficulty was due to the skin around her clitoris and proposed a clitoral hood reduction in addition to the labiaplasty. Given the well-documented orgasm gap in heterosexual relationships (Frederick et al., 2018), along with P5’s indication that she had no issues climaxing solo and her comment about the varying sexual skills of partners (“one man is not the next”), it seems more likely that her difficulties stem from partner dynamics rather than anatomical issues. The doctor’s suggestion that surgery could improve sexual function is questionable, especially considering the risks of surgery, such as potential pain, scarring, or loss of sensitivity.

In contrast, the approach of P6’s gynecologist shows how sensitive, informed counseling can both reassure patients and support well-considered decisions.

“[My gynecologist] told me: ‘You are absolutely abnormal. Everyone is different. Some have short labia, some long, some thick, some thin.’ She really reassured me that it was nothing abnormal.” (P6, 25).

Nonetheless, the concern lingered, so P6 asked about her options. The gynecologist provided information about plastic surgery but encouraged careful reflection, instituting a compulsory three-month reflection period:

“She said: ‘With this information, I’m going to let you reflect for a while on your choice. Know that surgery has an impact; you’ll need time to recover.’ At that point, I had already made up my mind that I wanted it, but she really wanted me to think it through again and reminded me, ‘Everyone is different.’” (P6, 25).

Looking back, P6 recognized how important that reassurance and reflection period had been:

“With a doctor, things can come across differently. If the doctor had said, ‘Ah yes, I see the problem,’ I would’ve thought, ‘Okay, there really is a problem, and I need the surgery now.’ But because she reassured me that there’s a lot of variation… In my case, it didn’t change my mind, but I can imagine it could convince someone else not to go through with the surgery” (P6, 25).

### Evaluating Surgery as a Coping Strategy

All six women who underwent labiaplasty said they were satisfied with their decision. Some felt relieved of physical discomfort; others were reassured by the aesthetic outcome. Yet when speaking in more depth, many revealed moments of doubt, lingering insecurities, or conditional acceptance.

Some participants described sudden doubt immediately after the surgery, mainly because of the intense pain. P5, for example, said that the severe pain made her question whether she had made the right choice. She emphasized that she would never have undergone the procedure for aesthetic reasons alone: “the pain is just not worth it.” This is notable because, although she framed her decision as motivated by physical discomfort, she also revealed that a new sexual partner’s comment— that he had "never seen such long labia"—had made her self-conscious and led her to reinterpret longstanding discomfort as something abnormal.

Other reflections, shared during the interviews, were more about decisions that felt not fully informed. P1 recalled feeling “really happy” with the result, especially because her “right inner labia now looked identical to the left one,” achieving a perfection she had strived for. Yet in hindsight, she expressed regret, not about the surgery itself, but about the underlying reasons. She reflected that earlier conversations with other women or exposure to vulval diversity might have changed her mind:

“I do feel it’s a bit of a shame that I did it, because I realize now: if I had talked to other women at that age, maybe there would have been more openness around this. And maybe then I wouldn’t have had the labiaplasty because of shame… Wait, let me put that clearly: maybe I wouldn’t have done it because of shame, but instead just because of the pain I had while cycling, or whatever else. But now I really feel like… I mostly did it because of shame” (P1, 36).

P2 described herself as “satisfied but not 100%” with the outcome: “one lip is still bigger than the other, and you can see that it curves slightly upwards at the bottom.” When asked whether anything about the surgical process could have been improved, she reflected that earlier exposure to body-positive projects like Hanne Lamon’s vulva photography might have shifted her perspective and decision-making:

“I really wonder if I would have gone through with the surgery if I had met Hanne [Lamon] earlier. The photoshoot would have been more difficult for me, but I do think it could have had a positive effect” (P2, 19).

P3 reported that the physical tension between her legs was gone after surgery, but she developed aesthetic concerns that hadn’t existed before, concerns she directly attributed to her surgeon’s warning that, without clitoral hood reduction, her clitoris might look “like a little penis.” Post-surgery, she felt dissatisfied with the transition between her labia and clitoral hood, describing it as an imperfect “little flap.”

P4, who had her labiaplasty at age 15, felt deeply relieved afterward, saying the procedure significantly reduced her insecurity. However, she also described a lingering desire to hide the fact that she had undergone surgery. Her previous partner never raised the issue—and hadn’t even noticed the surgery—but now, anticipating the gaze of a more sexually experienced partner, her insecurity was creeping back in. This suggests that, for her, the surgery didn’t fully resolve the deeper question: Is my vulva acceptable? During the interview, she expressed surprise when she learned that around half of all women have inner labia that protrude beyond the outer ones:

“Oh! I didn’t know that until now… Maybe that would’ve made a difference”

Still, she added that she likely would have proceeded with the surgery anyway, especially after the surgeon described her case as “severe.” When asked whether a more reassuring medical response might have changed her mind, she acknowledged that it might have, before quickly adding: “Then I probably would’ve just gone to another doctor to ask again.” Her comments reflect a subtle tension between the weight of external validation and her own sense of urgency at the time, as well as the enduring impact of not having had access to basic information earlier.

P6, who was the only participant to receive clear reassurance, balanced information, and a mandatory reflection period from her gynecologist, was also the only one who expressed full, unambiguous satisfaction with no lingering doubts.

## Discussion

This study confirms what previous research has shown: genital insecurities are widespread among cisgender women and often carry consequences for self-esteem, sexuality, and everyday life. However, two dynamics deserve explicit attention. First, insecurities were strongly shaped by imagined others. Participants’ anxieties often stemmed less from direct criticism than from anticipating how others, especially male sexual partners, might view them. Genital self-image thus emerged not as a private evaluation but as a relational, socially negotiated phenomenon. This dynamic aligns with symbolic interactionist accounts of identity as formed through others’ eyes (Goffman, 2022) and with objectification theory (Fredrickson & Roberts, 1997), which highlights how the male gaze structures women’s self-perception and bodily practices.

Second, I introduce the concept of insecurity creep to capture how genital concerns accumulate and expand over time. Participants rarely described all their insecurities arising at once; instead, new worries emerged incrementally, often when they learned that yet another feature could be framed as problematic. Remarks from peers or advertisements for new beauty practices (such as labiaplasty or anal bleaching) led women to ask: “Should I be worried about this too?” This mechanism shows how insecurities spread not only through direct criticism but through cultural cues and market prompts. Insecurity creep connects these lived experiences to broader feminist analyses of the intensification and extensification of beauty norms (Gill, 2021): as more body parts come under scrutiny and standards grow ever stricter, women’s insecurities expand to match.

How did women live with and respond to these insecurities? Coping unfolded along a continuum. Some managed their feelings through silence or avoidance, while others described gradual easing over time through age, experience, or peer conversations. Still others actively reframed their relationships with their vulvas, drawing on body-positive media, art projects, or queer sexual cultures that celebrated diversity. Surgery appeared within this continuum as one strategy, but not the only one. This underscores the need to situate female genital cosmetic procedures within a broader landscape of practices through which women negotiate insecurity.

Within this landscape, medical encounters played a rather ambiguous role. Participants rarely experienced clinicians as sources of reassurance. Many did not feel comfortable asking whether their genitalia were “normal,” and when they did, responses often reinforced insecurity. Particularly concerning were cases where doctors raised concerns unprompted or framed natural variation as “excess skin” or “labial hypertrophy.” Such interactions reflect the process of medicalization described by Braun (2019), in which aesthetic variation is pathologized and surgery presented as a corrective. While this may partly reflect clinicians’ efforts to validate women’s concerns in a context where women’s complaints are often dismissed, it risks transforming insecurity into medical “problems,” making surgery appear necessary rather than optional.

Not all women, however, sought surgery because they believed they were abnormal. Some pursued it despite recognizing their vulvas as within the range of normal variation, framing their decision instead as enhancement or relief from minor discomfort. At first glance, this reflects what Conrad (2005) terms a “second wave” of medicalization, where medicine shifts from curing illness to optimizing bodies. Yet the accounts here suggest something more: optimization was not always patient-led. Clinicians themselves sometimes introduced additional procedures—such as clitoral hood reduction—by invoking new aesthetic worries or promises of enhanced orgasm. In doing so, they did not merely respond to women’s pre-existing concerns but actively expanded them. This points to a clinical role in the intensification and extensification of beauty norms (Gill, 2021): physicians, drawing on their authority, help to translate shifting cultural ideals into medical needs, extending the reach of the beauty system into the consultation room.

Finally, while all the women who underwent labiaplasty described themselves as satisfied and some said they would recommend the procedure, their narratives revealed important complexities. Relief often coexisted with lingering doubt, regret, or new concerns. Several questioned their motivations in hindsight, speculated about what might have changed their minds, or worried about how future partners might respond. This suggests that “satisfaction” is not a straightforward outcome but a layered, ambivalent experience, something often missed in surveys that reduce it to a single measure.

Notably, the only participant who reported unambiguous satisfaction (P6) was also the only one who experienced a clinical encounter that combined reassurance about genital diversity with clear information on risks and a mandatory reflection period. This contrast highlights how medical encounters shape not only whether women choose surgery, but also how they live with that choice afterward. Even when women proceeded with FGCP, their sense of confidence or doubt hinged on how their anatomy and concerns were framed: as a “problem” in need of correction, or as a variation of normality. When clinicians offered a combination of reassurance, information, and time for reflection, women described greater acceptance and confidence. When encounters medicalized variation and rushed toward correction, satisfaction was more fragile, shadowed by doubts or new insecurities, especially when women later learned that their anatomy had always been within the range of normal.

### Conclusion and Implications

Taken together, this study shows that genital insecurities among cisgender women are not simply private dissatisfactions but socially shaped, relational experiences that often intensify through cultural cues and clinical framings. Women respond along a continuum of coping strategies, from silence and avoidance to reframing and, in some cases, surgery. Crucially, medical encounters can emerge as a decisive juncture: they can deepen insecurity when variation is pathologized, or foster lasting confidence when diversity is normalized and decisions are supported with balanced, sensitive counseling. By tracing these dynamics, the study contributes to feminist debates on the intensification of beauty norms and highlights the need to understand genital cosmetic procedures as embedded within broader cultural and clinical contexts.

These findings carry several important implications for education, cultural discourse, and clinical practice. First, they underscore the urgent need for comprehensive sex education that includes information about genital diversity, counters shame, and equips people to navigate unrealistic cultural ideals. This should begin with school-based education, but can be strengthened by broader cultural interventions. Media campaigns and body-positive art projects, such as The Vulva Gallery and Hanne Lamon’s Vulva Obscura project, play a crucial role in challenging narrow beauty norms and affirming the normalcy of vulval diversity.

Second, educational efforts must extend to medical professionals. As perceived arbiters of “normality,” clinicians are well placed to counter misinformation and support informed decision-making. Training should emphasize both anatomical diversity and sensitive communication: reassuring patients about variation, avoiding pathologizing language (“problem,” “labial hypertrophy,” “excess”), and providing balanced information about surgical and non-surgical options, including risks. Respecting autonomy does not mean endorsing surgery uncritically; it means creating space for accurate information, compassionate dialogue, and genuine reflection. While mandatory reflection periods have been criticized in feminist debates—particularly in the context of abortion—for potentially undermining autonomy, it remains essential that decisions about labiaplasty are not rushed. Encouraging patients to take their time, ask further questions, and revisit their decision without pressure (such as persuasive follow-up calls) can help ensure that consent is genuinely informed.

## Data Availability

Due to the sensitive and personal nature of the data, interview and focus group transcripts are not publicly available. Anonymized excerpts are available from the author upon reasonable request and in accordance with ethical guidelines.

## Footnotes

1 FGCP are distinct from gender-affirming surgeries sought by transgender individuals to align their body with their gender identity. They are also distinct from surgical interventions performed on intersex children, which are carried out without consent to enforce normative gendered appearance. Although FGCP technically meet the World Health Organization’s definition of female genital mutilation/cutting (FGM/C)—as any injury to female genitalia for non-medical reasons—they are rarely categorized as such. The term FGM/C is typically reserved for practices in African and Asian contexts, a distinction that has been criticized for reinforcing racist and colonial assumptions about consent, agency, and victimhood in Western vs. non-Western women (Ahmadu, 2017; Van Bavel et al., 2025).

2 “Schaamstreek” (pubic region) and “schaamhaar” (pubic hair) are used for people of any gender. “Schaamlippen” (“shame lips”) refers only to the labia, with no equivalent “shame” terminology applied to male genitals.
